# KLHL40-Related Myopathy: A Systematic Review and Insight into a Follow-up Biomarker via a New Case Report

**DOI:** 10.3390/genes15020208

**Published:** 2024-02-05

**Authors:** Bianca Buchignani, Gemma Marinella, Rosa Pasquariello, Giada Sgherri, Silvia Frosini, Filippo Maria Santorelli, Alessandro Orsini, Roberta Battini, Guja Astrea

**Affiliations:** 1 Department of Neuroscience, IRCCS Stella Maris Foundation, 56128 Pisa, Italy; bianca.buchignani@fsm.unipi.it (B.B.); gemma.marinella@fsm.unipi.it (G.M.); rosa.pasquariello@fsm.unipi.it (R.P.); giada.sgherri@fsm.unipi.it (G.S.); silvia.frosini@fsm.unipi.it (S.F.); guja.astrea@fsm.unipi.it (G.A.); 2 Department of Translational Research and of New Surgical and Medical Technologies, University of Pisa, 56126 Pisa, Italy; 3 Molecular Medicine and Neurogenetics Unit, IRCCS Stella Maris Foundation, 56128 Pisa, Italy; filippomaria.santorelli@fsm.unipi.it; 4 Pediatric Neurology, Azienda Ospedaliera Universitaria Pisana, 56100 Pisa, Italy; aorsini.md@gmail.com; 5 Department of Clinical and Experimental Medicine, University of Pisa, 56126 Pisa, Italy

**Keywords:** congenital myopathy, *KLHL40*, nemaline myopathies, KLHL40-related myopathy

## Abstract

Background: Mutations in the *KLHL40* gene are a common cause of severe or even lethal nemaline myopathy. Some cases with mild forms have been described, although the cases are still anecdotal. The aim of this paper was to systematically review the cases described in the literature and to describe a 12-year clinical and imaging follow-up in an Italian patient with KLHL40- related myopathy in order to suggest possible follow-up measurements. Methods: Having searched through three electronic databases (PubMed, Scopus, and EBSCO), 18 articles describing 65 patients with homozygous or compound heterozygous *KLHL40* mutations were selected. A patient with a *KLHL40* homozygous mutation (c.1582G>A/p.E528K) was added and clinical and genetic data were collected. Results: The most common mutation identified in our systematic review was the (c.1516A>C) followed by the (c.1582G>A). In our review, 60% percent of the patients died within the first 4 years of life. Clinical features were similar across the sample. Unfortunately, however, there is no record of the natural history data in the surviving patients. The 12-year follow-up of our patient revealed a slow improvement in her clinical course, identifying muscle MRI as the only possible marker of disease progression. Conclusions: Due to its clinical and genotype homogeneity, KLHL40-related myopathy may be a condition that would greatly benefit from the development of new gene therapies; muscle MRI could be a good biomarker to monitor disease progression.

## 1. Introduction

Nemaline myopathy (NEM) is a rare congenital genetic muscle disorder that varies in clinical severity presentation and clinical onset, but it is histologically determined by the presence of inclusions in the cytoplasm known as nemaline bodies or rods in the muscle biopsy [1,2].

The clinical picture is variable. Clinical severity may range from moderate muscular problems that can begin in childhood or later-onset myopathy, which occurs in adulthood, to severe forms with early mortality. The muscle weakness distribution can also be variable, ranging from distal to generalized, and it usually improves slowly [3]. Moreover, there might also be a variable onset with severe muscle weakness already in utero. NEM can be caused by mutations in 14 different genes, including ACTA1, NEB, KLHL40, TPM2, TPM3, CFL2, TNNT1, LMOD3, KBTBD13, MYPN, TNNT3, RYR3, KLHL41, CAP2, and MYO18B. All these genes encode proteins associated with the structure or regulation of the thin filament of the skeletal muscle sarcomere. Among these, NEB and ACTA1, which are responsible for encoding actin α 1 (ACTA1) and nebulin (NEB) proteins, respectively, which in turn are integral components of the sarcomeric thin filament, are the most commonly associated genes. Specifically, it is estimated that mutations in the NEB gene contribute to over 50% of NEM cases, while mutations in the ACTA1 gene are responsible for 15–25% of cases [4].

Diagnosis usually relies on clinical suspicion and weakness patterns. In the congenital onset, diagnosis is guided by the evidence of severe hypotonia, proximal muscle weakness, arthrogryposis, severe respiratory insufficiency, and feeding problems. Diagnosis is supported by paraclinical studies, such as creatinine kinase levels, potentially showing normal or slightly elevated results, and electromyography, revealing nonspecific myopathic changes. The increasing trend is for a diagnosis to be established or confirmed through molecular genetic studies, which concentrate on mutations in genes linked to the disease, thanks to advancements in genetic tools. Histopathological confirmation is achieved through the analysis of a muscle biopsy, where characteristic rod bodies (nemaline bodies) are identified in the sarcoplasm and highlighted by a modified Gomori stain [5].

Moreover, it is of interest to note that in the last decade, many diagnoses have been performed in utero thanks to the use of an NGS panel and prenatal ultrasound, which can detect clues that lead to the diagnosis of NEM, such as reduced or absent fetal movements, polyhydramnios, and extremity anomalies [6].

Currently, there is no curative therapy for NEM. Care of the patient focuses on managing symptoms, which includes preserving joint and muscular mobility, keeping an eye on respiratory function, providing treatments when necessary, and gaining independence in everyday activities.

With regard to muscle magnetic resonance imaging (MRI), a recent review of MRI findings highlighted involvement of the tibialis anterior and soleus muscles in the most common phenotypes (NEB-NEM, TPM3-NEM, ACTA1-NEM). Among these, the tibialis posterior was always involved in TPM3-NEM but was spared in the other two genotypes. Less is known about KLHL40-NEM [7].

Among the 14 genes that can cause NEM myopathies, a mutation in *KLHL40* causes NEM 8 (OMIM #615340), which is one of the severe forms of NEM [8]. Nineteen mutations (comprising four frameshifts, twelve missense mutations, two nonsense mutations, and one splice site) in the *KLHL40* gene were identified for the first time in 2016. These mutations were found in 28 apparently unrelated families within a cohort of 143 families affected by severe NEM [8].

*KLHL40* provides instructions for producing a protein called Kelch-like 40 (KLHL40). KLHL40 protein belongs to the superfamily of Kelch-repeat-containing proteins, striated-muscle-specific proteins that form characteristic β-propeller structures involved in muscle development and function [9].

The incidence of NEM8 is variable in different countries. However, it has a higher incidence in the Chinese population, while some mutations are recurrent in Turkey and Japan, suggesting a founder mutation effect in those populations [8,10,11,12,13,14].

The onset of the disease can vary, yet it is usually congenital. However, some clinical signs may be detected during pregnancy. In particular, fetal akinesia/hypokinesia or polyhydramnios are described in the majority of patients [15]. Moreover, during the neonatal period, most children suffer from respiratory failure, and death can occur before the child reaches 6 months of age [8].

Unfortunately, as in NEM myopathies, there is currently no effective treatment available for patients with *KLHL40* mutations. However, there have been reports of a female patient with KLHL40-related NEM experiencing a significant and sustained improvement following treatment with acetylcholinesterase inhibitors [16].

The aim of this review was to describe the cases of KLHL40-related myopathy thus far reported in the literature, including a girl harboring a homozygous mutation (c.1582G>A/p.E528K in *KLHL40*) who has been followed at our Institute for 12 years. The secondary aim was to suggest possible clinical outcome measures and biomarkers for future natural history studies.

## 2. Materials and Methods

### 2.1. Search Strategy and Selection Criteria

**Figure 1:**
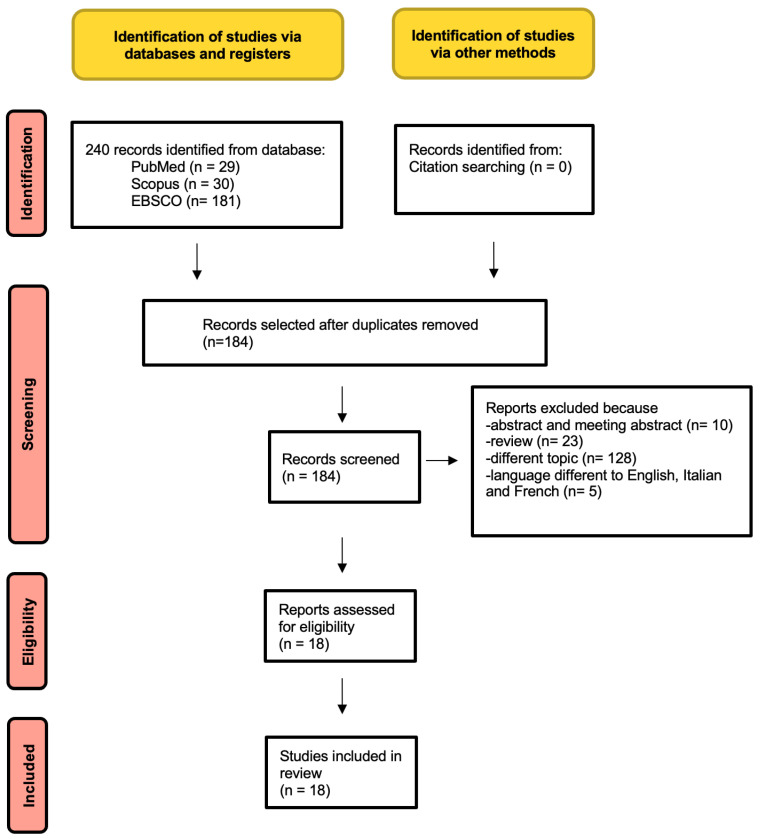
PRISMA flowchart of review.

A systematic search strategy was conducted following the guidelines of Preferred Reporting Items for Systematic Reviews and Meta-Analyses (PRISMA) (Figure 1) [17].

The literature search was carried out using three electronic databases: PubMed (https://pubmed.ncbi.nlm.nih.gov/, accessed on 27 January 2023), Scopus (https://www.scopus.com/, accessed on 27 January 2023), and EBSCO (https://www.scopus.com/, accessed on 27 January 2023). The search strategy, designed to include all fields (such as titles, abstracts, and keywords), used strings adapted for each database: (“KLHL40” AND “nemaline myopathy”). We carefully searched the reference lists of the original studies and reviews identified by the search in order to identify additional studies meeting our criteria. We used the research tool “Zotero” (https://www.zotero.org/download/, accessed on 27 January 2023) to collect all the results in a single library.

The inclusion criteria used to select articles were the following: (i) patients with congenital myopathy and (ii) a description of the presence of homozygous or compound heterozygous mutations in *KLHL40*. The exclusion criteria were (i) duplicates; (ii) studies not concerning the aim of the paper; (iii) articles written in languages other than English, Italian, and French; and (iv) reports in the form of abstract, review, theses, and conference papers.

The full list of all potentially eligible articles were obtained by two authors (G.M. and B.B.) working independently. Any disagreements were resolved through consensus or, when necessary, by a third reviewer (G.A.).

The PRISMA flowchart shows the process of identification and selection of papers: 240 abstracts were initially retrieved. Once duplicates had been removed, 184 records were screened, 10 of which were excluded because they were abstract, 23 were excluded because they were reviews, 128 were excluded because they were on a different topic, and 5 were excluded because they were written in a language other than English, Italian, and French. Finally, 18 studies were included in the systematic review (Figure 1).

Seventy patients were described in the 18 selected studies; 65 were selected once the duplicates had been removed.

### 2.2. Data Collection Process

Data were collected on the genetic, clinical, muscle biopsy, and muscle imaging features of the patients reported in each of the included studies. In particular, for each patient, we collected data on the type of genetic mutation, neonatal history and symptoms, contracture and fractures at birth, the age and symptoms of the patient at the last evaluation, muscle weakness, facial involvement, dysphagia, muscle MRI, and muscle biopsy. If the data were not listed in the description, we wrote not applicable (NA).

### 2.3. Genetic Analysis

**Figure 2:**
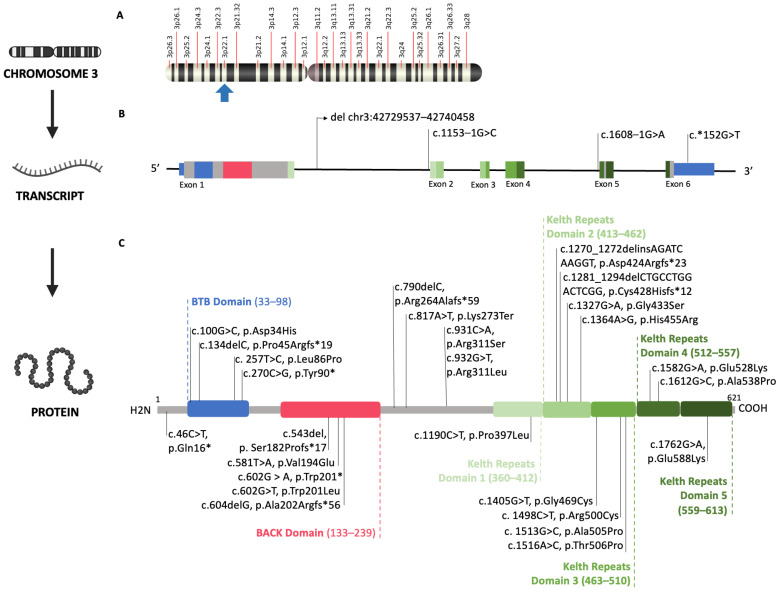
The *KLHL40* gene maps to chromosome 3q22.1 (**A**)-blue arrow). KLHL40 transcript contains 6 exons (**B**) and encodes the 621-amino acid KLHL40 protein, which has an N-terminal BTB and BACK domains (blue box and red box) and 5 C-terminal Kelch repeats (green boxes) that are predicted to form a β-propeller structure (**C**). The corresponding protein domains are represented according to the Protein Families (PFAM) database (**C**). Schematic view of the *KLHL40* mutations of patients described in the present work with related position according to UNIPROT (https://www.uniprot.org/uniprotkb/Q5EB39/entry, accessed on 23 January 2024).

Each variant of the patients was studied to identify its position in the human *KLHL40* gene sequence with Varsome (https://varsome.com/, accessed on 23 January 2024) [18] and the protein structure with Uniprot (https://www.uniprot.org/, accessed on 23 January 2024) [19]. In Figure 2, the graphic representation of these data is reported (Figure 2).

The mutations in our patient were detected in our genetic laboratory using a multiexon amplicon panel containing a total of 241 genes known to be associated with muscular dystrophies and myopathies. The data were analyzed and prioritized using bioinformatic tools and modalities already reported elsewhere [20]. No other variants, except the KLHL40 reported, were pathogenic/likely pathogenic and segregated in family members.

### 2.4. Case Report

We report the case of a 13-year-old Italian girl, the second child of healthy nonconsanguineous parents, with a homozygous c.1582G>A/p.E528K mutation in the *KLHL40* gene.

She was born at 38 weeks by cesarean section due to a podalic presentation. During pregnancy, her mother presented mild placental abruption in the second month of gestation and gestational diabetes. Fetal movements were reported as being less valid than in her previous pregnancy, while fetal growth was in the normal range. At birth, she weighed 2950 g and presented with bilateral generalized arthrogryposis, femoral fracture, severe hypotonia, and ventilatory distress. Due to the severity of the clinical features, she was hospitalized in the neonatal intensive care unit, and owing to the presence of severe dysphagia, nasogastric feeding was started. At the age of 11 months, she underwent surgery to reconstruct her esophageal hiatus and a percutaneous endoscopic gastrostomy (PEG) was positioned. A muscle biopsy was performed during the surgery but was found to be poorly contributory due to technical artifacts. In order to exclude the hypothesis of congenital myasthenia, pyridostigmine bromide was started at the age of 1 month (at 4 mg/Kg/die) but was suspended by the age of one year due to inefficacy.

When she was first referred to our Institute at 5 months of age, she presented proximal and axial weakness, hypotonia, laxity, syndactyly, and myopathic facies with hypomimia. Her deep tendon reflexes were weak. An early intensive physiotherapy program helped to improve her motor skills and reduced limb arthrogryposis.

At 9 months of age, head control was acquired, and neurodevelopmental examinations performed with the Bayley III neurodevelopmental scale showed a result at 37° percentile for age (score in the low normal range for age). The sitting position was acquired at 15 months and she began walking at 38 months. As her language development was delayed, she started speech therapy at the age of three and showed some improvement. By the age of four, she produced some dysarthric sentences. Her motor abilities increased over time. Motor functional assessments were performed during follow-up visits (Motor Function Measure (MFM) [21]) to monitor the disease progression; a spirometry was also performed (see Table 1).

She presented mild dorso-lumbar scoliosis from the age of 4, which remained stable until she was 10 years old. When she was 12, the scoliosis evolved to a Cobb angle of 22.5 degrees, and a Cheneau brace was introduced. Over the last 18 months, the curve has increased, with a Cobb angle changing from 22.5 to 37 degrees, concomitant with the pubertal growth spurt. At the age of nine, hypostaturalism due to GH deficit was detected. She presented frequent respiratory infections (rhinitis/rhinopharyngitis, bronchitis, and one episode of pneumonia at the age of 2), and a cough machine was introduced. She has performed regular pulmonary function tests, which have shown results in the normal range since the age of eight. Currently, she is still using the cough machine daily, but no ventilation support is needed at night. Dysphagia has reduced over time, but at present, feeding takes place both orally and through a PEG tube.

The genetic diagnosis was reached by the age of 7 using an NGS panel containing multiple genes associated with congenital myopathies, which revealed a homozygous variant in *KLHL40* (c.1582G>A/p.E528K) [22].

**Figure 3:**
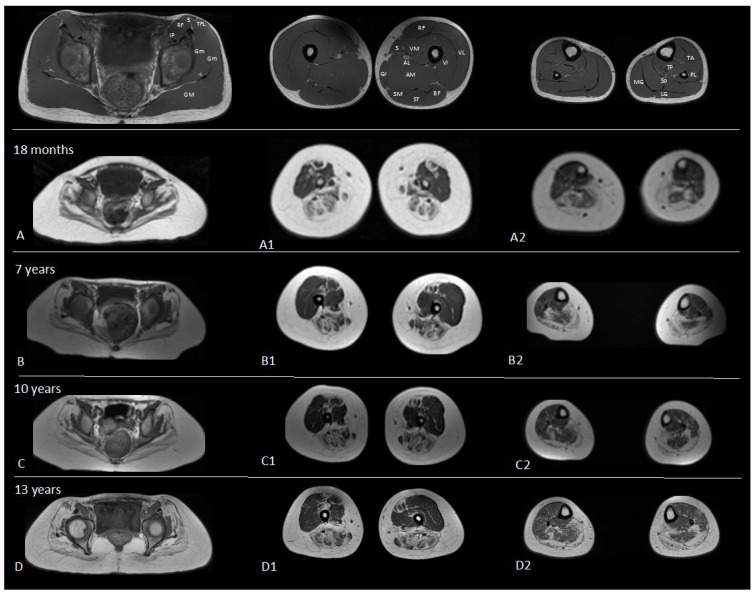
Sequential imaging studies show slow progression over time. (**A**–**D**). The axial T1 weighted image at 18 months shows: marked fat infiltration and hypotrophy of the gluteus muscles (maximum(GM), medius (Gmed) and minimus gluteus(Gmin)) (**A**); at thigh level (**A1**) is evident the prevalent involvement of the posterior compartment (biceps femoris muscle (BF), semitendinosus (SM), semimembranosus (SM)) and of medial muscles (adductor longus muscle (ADL), adductor magnus muscle (ADM) and gracilis muscle (Gr)) with complete fat replacement of the sartorius. A modest fibro-adipose infiltration of the mid-distal sections of the rectus femoris muscle is evident too (**A1**). At leg level (**A2**) complete fat replacement of the gastrocnemius muscles and the peripheral portion of the soleus muscle can be seen, with relative sparing of anterior compartment. This pattern remained stable in two consecutive examinations (7 years (**B**,**B1**,**B2**) and 10 years (**C**,**C1**,**C2**)) and it showed only minimal variations at 13 years (**D**,**D1**,**D2**), with increased involvement of the vastus intermedius (**D1**) and the soleus (**D2**).

During follow-up, four thigh and leg muscle magnetic resonance imaging (MRI) tests were conducted (at 18 months and when she was 8, 11, and 13 years old). The first three were performed with a 1.5T GE scanner, while a 3T GE scanner was used for the latter. From the very first examinations conducted, the images revealed a major involvement of the paraspinal and glutei muscles, which showed fat replacement and hypotrophy. The most involved muscle compartment of the thigh was the lateral posterior area, where the biceps femoris was completely replaced by fatty infiltration. All the exams showed a relative sparing of the anterior compartment except for the rectus femoris. This initially showed a peripherical circular area with fatty replacement (target type), which, however, gradually evolved into a complete replacement over time. By the third examination, the sartorius muscle had been completely replaced. The images of her legs also showed a mild adipose infiltration of the posterior compartment with a very slight involvement of the peronei group and sparing with relative hypertrophy of the tibialis posterior muscle (Figure 3).

Currently, the girl is 13 years old and still has mild axial and proximal weakness, mostly in her lower limbs. She can walk with marked hyperlodosis and climb the stairs with support, but she cannot run. Her scoliosis, which showed a Cobb angle of 37 degrees at her last evaluation, has not interfered with her ability to walk, even for long distances. Her cognitive abilities are preserved.

## 3. Results

### Descriptive Findings

Eighteen studies, reporting a total of 70 patients, were included in this systematic review. After removing the duplicates, a total of 65 patients were included. Please see the Supplementary Materials, Table S1, for the description of the patients. In the analyses, we have also included the additional patient described in the present case report.

The 65 patients included in the review present a total of 30 different genetic alterations. Among these, four affect the BTB domain, five impact the BACK domain, twelve involve Kelch repeats domains, and seven occur within interdomains. Lastly, one alteration is a deletion encompassing exons 2–6 of the *KLHL40* gene and one alteration occur within 3′-untranslated region (3′UTR).

Of the 65 patients described, 15 harbored the same homozygous mutation (c.1582G>A) as our patient, whereas two patients presented the same variant in a compound heterozygosis. The most common mutation in the overall sample was the (c.1516A>C), accounting for a total of 35/130 (27%) alleles.

In 12 of the 65 cases, an intrauterine death had occurred (18%); 22 infants died before the first year of life (34%), and 5 children before the age of 4 (8%). Of the remaining sample, the age at description varied and ranged from 1 month to 20 years, with a mean of 2.36 years ± 5.56. Only two patients were older than our patient and had different mutations (see Supplementary Material, Table S1). No genotype–phenotype correlation was found with increasing age nor were milder phenotypes found.

In fact, as reported in Figure 2, patients with the c.1582G>A mutation in homozygosis may experience an early death or may survive up to 11 years. The factor that most influences the severity of the clinical picture and affects survival seems to be the degree of weakness at birth. Of the 28 patients described with fetal akinesia, only 3 survived the neonatal period, and there is no information available regarding one of them. Of these 28 patients, 7 present the c.1516A>C variant in homozygous and 5 in heterozygous, but the other 16 present variants spread across the entire length of the gene.

With regard to the nationality of those collected in our study, 21 individuals were Chinese, 14 were Japanese, and 9 were Turkish, while the remaining were of different nationalities. Only one other case was Italian. Twenty-seven patients were female and 28 were male; the sex had not been specified in 10 cases. Twenty-eight patients had a family history of *KLHL40*-related myopathy (43%). The myopathy onset was antenatal in the majority of the patients with early signs being detected during pregnancy (76%); fetal akinesia or hypokinesia was described in 58% of the cases. Of the 54 infants alive at birth, 13 (24%) were born before 37 weeks of gestational age. A total of 41 infants (76%) presented contractures at birth and 19 (35%) had fractures.

Clinical features were similar across the sample and were described in 50/53 patients. Muscle weakness was described in all the patients; 28/50 (56%) were not able to move, and 14/50 (28%) presented severe weakness, while the remaining 8 (16%) presented mild weakness. Asphyxia or respiratory failure was described in almost all the samples, with three exceptions. In 28/50 (56%) cases, ventilatory support was required. Dysphagia was also a predominant sign, being described in 36 patients, requiring tube-feeding or gastrostomy in 25 infants. A total of 11 patients underwent a muscle biopsy and 3 a muscle MRI. None of the patients, except our patient, had a follow-up MRI. Other features described were chest deformities in 15/50 patients (3%), scoliosis in 4/50 patients (8%), brain abnormalities in 4/50 children (8%), microgenitalia in 4 infants (8%), and cardiac defects in 3/50 cases (6%).

GH deficiency, which had not been previously described in the literature, has been outlined in our case description.

With regard to muscle MRI imaging, only two case reports included the description of the scans. In the first, the boy showed wide fat replacement of the pelvic girdle muscles of the posterior compartments of the thighs except for the involvement of the rectus femoris and of the lower legs [23]. In the other, the girl’s muscle MRI, which was performed when she was 9 years old, was normal [24].

## 4. Discussion

Nemaline myopathy due to mutations in *KLHL40* is the most common form of severe nemaline myopathy and represents a rather homogeneous entity from both a clinical and genetic perspective.

As reported in the pertinent literature, common prenatal features included reduced fetal movement, polyhydramnios, and clubfeet, while contractures, respiratory failure, and swallowing difficulties are described at birth. Muscle weakness is usually severe, and almost half of the individuals have no spontaneous antigravity movement [1]. The survival rate of those in our study ranged from 49 days to 17 months. Respiratory failure and respiratory infections are documented as being the main causes of death and are also present in approximately 60% of the patients by the age of 4 [25].

Postnatal features are similar to those of other congenital nemaline myopathies [26].

In an attempt to identify markers that could assist the clinician in predicting the severity of the phenotype or define, at an early stage, cases that presented a milder clinical course, we also tried to establish more precise genotype–phenotype correlations on the basis of the data in the literature (see Figure 2). However, this correlation appears weak, and it is necessary to expand the study to a larger sample size upon the identification of new moderately affected cases.

The c.1516A>C variant appears to be the most common variant, being detected in approximately one-third of the patients described in the literature either in homozygosis (11 patients) or in compound heterozygosis (12 patients). A Chinese-specific founder mutation effect has been recently reported [25], and prenatal screening in Chinese fetuses with features that are suggestive of congenital neuromuscular disorders, such as prenatal symptoms and/or contractures or with a significant family history of congenital myopathies, has been suggested.

Congenital myopathies are often rare and heterogeneous conditions with various phenotypes and a complex defective protein structure and function. This characteristic often influences their suitability for different therapeutic strategies. Despite this, therapeutic attempts have been made in some forms of myopathies with nongenetic drugs, which intervene to modulate some metabolic alterations that occur within the muscle fiber (i.e., salbutamol) or at the neuromuscular junction level [27].

Recently, it has been reported that a patient with KLHL40-related myopathy due to the c.604delG/p.Ala202Argfs*56 and c.1513G>C/p.Ala505Pro mutations responded extremely well, and for a prolonged period, to a high dose of pyridostigmine (20 mg/kg/day). After 3 days, she showed a remarkable improvement in ventilation, and after 2 weeks, spontaneous movements had increased significantly; her facial expression had also improved. In view of this response, the patient was initially diagnosed as suffering from congenital myasthenia, and two mutations in *DOK7* were detected. However, in a later clinical reassessment, the detection of rods in a second muscle biopsy and further genetic investigations led to the conclusive diagnosis of *KLHL40*-related nemaline myopathies [16]. Hence, pyridostigmine was also initially administered and tested in our patient, albeit using a lower dose of the drug, but we did not observe any significant clinical improvement. Her development did progress, however, but certainly at a slower rate than that described in the patient reported by Natera-de Benito. We cannot exclude that the co-occurrence of variants in *DOK7*, though found commonly in polymorphic databases, exerts a yet undefined modulation of response to therapy. In our case, multigene testing did not disclose any additional variants in the genes associated with myasthenia. We can hypothesize that the poor response to treatment was related to the dose or lack of gene modifiers or even to other factors as yet unknown. However, considering the absence of severe or moderate side effects, the use of pyridistogymn in *KLHL40*-related nemaline myopathies should be proposed.

From a genetic point of view, we highlight that NEM8 in 70 percent of cases is caused by two common mutations (the c.1582G>A and the c.1516A>C). Due to an unclear genotype–phenotype correlation, it is possible to hypothesize that other genetic modulators or other factors, and not the mutation alone, may intervene in modulating the clinical phenotype.

In an era of new gene editing approaches and in times when finding therapies for rare inherited diseases is a health-related goal in Europe, the *KLHL40*-related nemaline myopathies may represent a good case for new examples of 1M1M development (https://www.1mutation1medicine.eu, accessed on 8 January 2024). In light of this, better work on how to measure and longitudinally assess NM8 should be promoted.

To date, only a few studies in the literature have investigated outcome measures in congenital myopathies. The Motor Function Measure (MFM), pulmonary function tests, and the slurp test were identified as promising outcome measures for clinical trials in a cross-sectional study of nemaline myopathy [26]. In another previous report, a Rasch-scaled MFM with only 25 items (Rs-MFM25 (CDM)) was identified as a valuable assessment tool for congenital muscle disorders [28].

In our case report, having used the MFM-32 scale, we report a mild improvement in motor function, as well as in the pulmonary function tests during follow-up using spirometry.

However, we also report the presence of progressive scoliosis, which started at the age of 4 and rapidly increased at the age of 10 due to the patient’s pubertal growth spurt. The young girl was also supported by GH therapy.

Based on the present systematic review, to the best of our knowledge, no follow-up data on the clinical course of the patients who have survived are available in the literature. One could hypothesize that the patients have shown a slowly improving clinical course, as described in other nemaline myopathies, but follow-up measures are needed.

**Figure 4:**
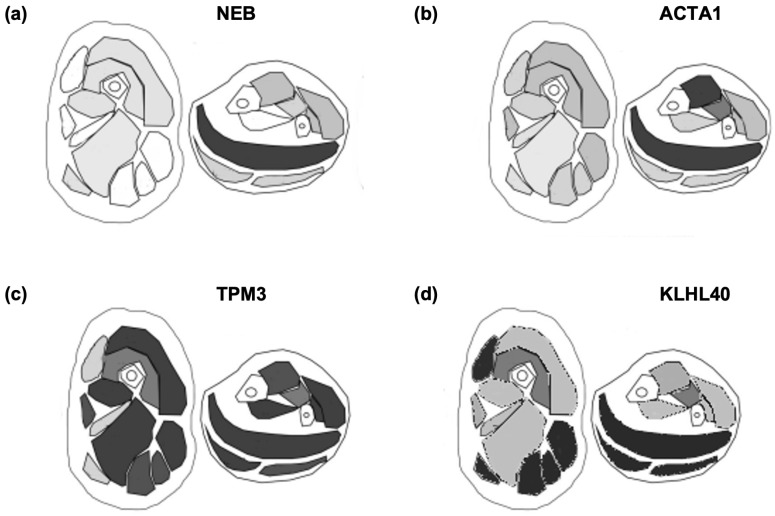
Overview of the muscle MRI pattern of NEM (Mod. by Perry et al. 2023 [7]). Gray scale where dark gray corresponds to the maximum degree of fibroadipose replacement. Note the different pattern present in NEM with prevalent involvement of soleus and tibialis anterior in NEB-NEM (Panel (**a**)) and in ACTA1-NEM (Panel (**b**)) and the prevalent involvement of the hamstring muscles in the thigh and comparative sparing of the rectus femori in TPM3-NEM (Panel (**c**)), while in KLHL40-NEM (Panel (**d**)), there is the prevalent involvement of hamstring muscles in the thigh but also of the rectus femori and the involvement of soleus and gastrocnemi with sparing of tibialis anterior.

With regard to muscle MRI, we report an involvement of the paraspinal and glutei muscles, an involvement of the lateral posterior muscle compartment of the thigh with a relative sparing of the anterior compartment, and of the posterior leg compartment with a sparing of the tibialis posterior. These data confirm and implement a previously reported MRI assessment (Figure 4).

Currently, the quantitative assessment of muscle fat fraction using Dixon sequences has been described as providing reliable and reproducible data with low inter- and intra-observer differences observed. Therefore, quantitative MRI analysis in research has indeed been demonstrated to be of prognostic value for several neuromuscular disorders [29,30,31]. Hence, for the severe forms, the main outcome measure is increased life expectancy; for those with a milder phenotype, we believe that muscle MRI represents the most reliable biomarker to assess progression [16,32]. The involvement of the posterior compartment of the leg is specific and prevalent, indeed, contrary to what happens in *NEB* mutations. Also, findings in *KLHL40*-related patients differ from other cases of NEM. Individuals having *TPM3*-related forms have a similar picture, though the rectus femoris is spared, whereas those with mutations in *ACTA1* show a typical involvement of the tibialis anterior muscle.

Finally, in this paper, we define distinct patterns of muscle involvement in NEM8 that may be useful both as a diagnostic and a progression biomarker. With regard to progression, in our case, we report muscle impairment, which was detected by muscle MRI, which showed a slight worsening over a 10-year period. Even though it is hard to speculate the long-term value of this tool in a longitudinal follow-up, the extent of fibroadipose replacement in muscle imaging could represent an accurate outcome measure for possible future gene therapy studies. Further studies on larger case series are warranted to confirm the validity of these findings to further evaluate genotype–phenotype correlations and to investigate the possible role of muscle MRI as a prognostic biomarker and outcome measure for these rare conditions.

## 5. Conclusions

In the last decade, the advent of new genetic analysis techniques has made it possible to not only establish an early diagnosis of a neuromuscular disease but has also increased the number of genetic diagnoses made in neuromuscular diseases, hence promoting the possible application of innovative gene therapies.

NEM8 is a recently recognized disease, which is still poorly studied. It is mostly diagnosed in certain ethnic groups, and it leads to premature death in approximately 50 percent of patients.

In this review and case report, we describe the second Italian case, which showed a mild improvement over time. As in other congenital myopathies, the use of a multidisciplinary clinical and rehabilitation approach can lead to an improvement both in life expectancy and in quality of life. However, in this case report, we highlight the importance of including a muscle MRI in the follow-up of congenital myopathies.

Even though muscle MRI pattern recognition has become a first-line complementary diagnostic tool in clinical practice in neuromuscular disorders, we emphasize the add-on value of a muscle MRI in the clinical follow-up to understand disease progression over time, and we highlight its possible role as a biomarker of progression in the hope that future therapeutic trials will be conducted soon.

## Figures and Tables

**Table 1:** Motor Function Measurement (MFM) scale scores and spirometry data.

	7 Years	11 Years	12 Years	13 Years
MFM total	77.08%	77.08%	78.13%	78.12%
D1 (standing position and transfers)	53.84%	51.28%	53.80%	51.28%
D2 (axial and proximal motor function)	94.44%	94.22%	91.67%	94.44%
D3 (distal motor function)	90.74%	95.23%	100%	100%
Spirometry				
FVC %/mL		93%/1710	83%/2030	85%/2340
PEF %/L/s		95/3460	80/3760	90/4540

## Data Availability

All procedures performed in this study were in accordance with the ethical standards of the institutional and/or national research committee and with the 1964 Helsinki Declaration and its later amendments or comparable ethical standards.

## Supplementary Materials

The following supporting information can be downloaded at: https://www.mdpi.com/article/10.3390/genes15020208/s1, Table S1: Summary of the laboratory and clinical features in the 65 patients with KLHL40-related myopathy [8,9,14,16,23,24,25,33,34,35,36,37,38,39,40,41,42,43].
