# Supplementary material for: KLHL40-Related Myopathy: A Systematic Review and Insight into a Follow-up Biomarker via a New Case Report
Source: Genes (Basel). 2024 Feb 5;15(2):208. doi: 10.3390/genes15020208 (PMC10887776; doi:10.3390/genes15020208)
Supplement: Supplementary file 1 [file genes-15-00208-s001.zip › genes-2850554-supplementary.pdf]

Table S1. Summary of the laboratory and clinical features in the 65 patients with KLHL40-related myopathy [8].

	Mutation	Nation	Sex	Age (Death)	Family History	Prenatal Symptoms	Fetal Akinesia/ Hypokinesia	Birth Age	BW	Asphyxia/ Respiratory Failure	Facial Weakness	Minor Dysmorphic Features	Dysphagia	Muscle Weakness	Contractures at Birth	Fractures at Birth	Chest Deformities	Cardiac Defect	Scoliosis	Brain Abn.	Micro-Genitalia
Ravenscroft et al. 2013[8]	c.134delC, p.Pro45Argfs *19 *homo	Italy	M	(2 m)	+	+	+	36	2735	++	++	NA	++	+++	+	+	-	-	-	-	-
	c.270C>G, p.Tyr90* *homo	Turkey	M	6 m	+	+	+	34	2500	++	++	NA	++	+++	+	+	+	+	-	-	+
	c.581T>A, p.Val194Glu *homo	Israel	M	(2y)	+++	+	+	41	3020	+	+	NA	++	++	+	-	+	-	-	-	-
	c.581T>A, p.Val194Glu *homo	Israel	M	18m	+++	+	+	32	1200	+	+	NA	++	++	NA	-	+	-	-	-	-
	c.602G>T, p.Trp201Leu *homo	Turkey	M	8y	+++	NA	NA	NA	NA	NA	NA	NA	NA	NA	NA	NA	-	-	-	-	-
	c.602G>T, p.Trp201Leu *homo	Norway	M	(9d)	-	+	+	36	1775	++	+	+	++	+++	+	-	-	-	-	-	-
	c.790delC, p.Arg264Ala fs*59 *homo	Turkey	M	(3d)	+++	+	NA	37	2130	+	NA	NA	NA	+++	+	NA	+	-	-	-	-
	c.932G>T, p.Arg311Leu ; c.1516A>C, p.Thr506Pro	China	M	(5d)	-	+	+	36	NA	++	+	+	+	+++	-	-	+	-	-	-	-
	c.1270_1272d elinsAGATC AAGGT, p.Asp424Arg fs*23; c.1582G>A, p.Glu528Lys	Japan	M	3y	-	+	+	34	2122	+	+	NA	+	++	+	+	+	-	-	+	-
	c.1281_1294d elCTGCCTG G ACTCGG, p.Cys428Hisf s*12; c.1582G>A, p.Glu528Lys	Korean	F	6m	-	+	NA	36	2646	+	+	+	+	+	+	NA	-	-	-	+	-
	c.1364A>G, p.His455Arg *homo	Turkey	M	(4m)	+++	-	-	41	3330	++	+	NA	++	+++	+	-	-	-	-	-	-
	c.1405G>T, p.Gly469Cys *homo	Japan	M	3m	-	+	NA	37	2412	+	++	+	+	++	+	+	+	-	-	-	+
	c.1405G>T, p.Gly469Cys; c.1582G>A, p.Glu528Lys	Japan	F	12m	+	+	+	38	2660	+	+	+	++	++	+	+	-	-	-	-	-
	c.1405G>T, p.Gly469Cys; c.1582G>A, p.Glu528Lys	Japan	M	(12 m)	+	+	NA	38	2500	+	+	+	++	++	+	-	-	-	-	-	-

	c.1582G>A, p.Glu528Lys *homo	Turkey	F	(3m)	+++	+	+	34	2590	++	+	+	++	+++	+	+	+	+	-	-	-
	c.1582G>A, p.Glu528Lys *homo	Turkey	F	(20d)	+++	+	+	37	2580	++	+	+	++	+++	+	+	+	-	-	-	-
	c.1582G>A, p.Glu528Lys *homo	Kurdistan	M	(10m)	+++	+	+	39	3380	+	+	+	++	+++	+	-	+	-	-	-	-
	c.1582G>A, p.Glu528Lys *homo	Kurdistan	M	11y	+	-	-	NA	NA	++	+	+	++	+	-	-	+	-	-	-	-
	c.1582G>A, p.Glu528Lys *homo	Japan	F	18m	NA	NA	NA	NA	NA	NA	NA	NA	NA	NA	NA	NA	NA	NA	NA	NA	NA
	c.1582G>A, p.Glu528Lys *homo	Japan	F	3m	NA	NA	NA	NA	NA	NA	NA	NA	NA	NA	NA	NA	NA	NA	NA	NA	NA
	c.1582G>A, p.Glu528Lys *homo	Japan	F	1m	-	-	-	37	2400	+	+	NA	+	++	NA	NA	NA	NA	NA	NA	NA
	c.1582G>A, p.Glu528Lys *homo	Japan	F	1m	NA	+	NA	40	2846	+	+	+	+	++	+	NA	NA	NA	NA	NA	NA
	c.1582G>A, p.Glu528Lys *homo	Japan	M	3m	+	+	NA	41	2410	+	++	NA	+	+	+	NA	+	-	-	-	-
	c.1582G>A, p.Glu528Lys *homo	Japan	F	6m	-	-	-	39	2505	+	+	NA	+	++	+	+	+	-	-	-	-
	c.1582G>A, p.Glu528Lys *homo	Japan	M	8m	-	+	NA	38	2640	+	NA	+	+	+	+	NA	-	-	+	-	+
	c.1582G>A, p.Glu528Lys *homo	Japan	F	10m	NA	+	+	38	3300	+	NA	NA	NA	+	+	NA	-	-	-	-	-
	c.1582G>A, p.Glu528Lys *homo	Japan	M	11m	-	-	-	42	2660	+	+	NA	+	++	+	NA	+	-	+	-	-
	c.1608–1G>A *homo	Turkey	M	(15d)	++	+	+	34	2030	++	+	NA	NA	+++	+	+	-	-	-	-	-
	c.1612G>C, p.Ala538Pro *homo	Turkey	F	(1h)	+++	+	NA	40	NA	+	NA	+	++	+++	+	NA	-	-	-	-	-
	c.100G>C, p.Asp34His; c. 257T>C, p.Leu86Pro	Vietnam	F	(30d)	+	+	+	38	2429	++	++	NA	NA	+++	+	+	+	-	-	+	-
	c.100G>C, p.Asp34His; c. 257T>C, p.Leu86Pro	Vietnam	M	NA	+	+	+	35	2515	++	NA	+	NA	+++	+	NA	-	-	-	-	+
	c.1190C>T, p.Pro397Leu; c.1762G>A, p.Glu588Lys	Turkey	F	20y	-	+	+	NA	3200	-	NA	NA	-	+	-	-	-	-	-	-	-
Todd et al, 2015[33]	c.46C>T, p.Gln16* *homo	NA	NA	(7w)	+++	+	+	35	1660	++	+	NA	+++	+++	+	+	-	-	-	-	-

	c.931C>A, p.Arg311Ser *homo	NA	NA	(2d)	+++	NA	NA	37	-	++	+	NA	NA	+++	-	NA	NA	NA	NA	NA	NA
Kawase et al., 2015[9]	c.1405G>T, p.Gly469Cys *homo	Japan	M	(4y)	-	-	-	37	2412	++	+	NA	NA	+++	-	+	-	-	-	+	-
D. Natera-de Benito, 2016[16]	c.604delG, p. Ala202Argfs *56; c. 1513G>C, p.Ala505Pro	Philippine	F	9y	-	+	+	39	2775	++	+	+	++	++	-	-	-	-	-	-	-
Tai-Heng Chen et al, 2016[34]	c.602G > A, p. Trp201*; c.1516A > C, p.Thr506Pro	Taiwan	NA	(21w)	+	+	+	NA	NA	NA	NA	NA	NA	NA	NA	NA	NA	NA	NA	NA	NA
	NA	Taiwan	NA	(28w)	+	+	+	NA	NA	NA	NA	NA	NA	NA	NA	NA	NA	NA	NA	NA	NA
	c.602G > A, p. Trp201*; c.1516A > C, p.Thr506Pro	Taiwan	F	(18w)	+	+	+	NA	NA	NA	NA	NA	NA	NA	NA	NA	NA	NA	NA	NA	NA
Seferian et al. 2016[24]	c.1498C>T, p.Arg500Cys *homo	Morocco	F	9y	-	-	-	39	3200	-	+	-	++	+	+	-	-	-	-	-	-
Waldrop et al. 2019[35]	c.932G > T, p.Arg311Leu *homo	NA	M	6m	NA	NA	+	NA	NA	++	++	NA	++	+++	+	-	-	-	-	-	-
Lee et al. 2019[14]	c.1516A>C, p.Thr506Pro *homo	China	F	(16m)	-	+	+	35	NA	++	+	-	++	+++	+	-	-	-	-	-	-
Avasthi et al. 2019[36]	c.42727712G >A, p.Trp201Ter *homo	NA	NA	NA	NA	++	++	NA	NA	NA	NA	NA	NA	NA	NA	NA	NA	NA	NA	NA	NA
Wu et al.,2020[37]	c.602G>A, p. Trp201*; c.1516A>C, p.Thr506Pro	China	F	(34w)	-	+	+	34	2145	++	+	NA	-	+++	+	-	-	-	-	-	-
Yi et al. 2020[38]	c.1153-1G>C; c.1516A>C, p.Thr506Pro	China	M	(1w)	+	+	+	37	2540	++	++	+	++	+++	+	-	-	-	-	-	-
	c.1153-1G>C; c.1516A>C, p.Thr506Pro	China	M	(1w)	+	+	+	37	2160	++	++	+	++	+++	+	-	-	-	-	-	-
Yeung et al. 2020[25]	c.1516A>C, p.Thr506Pro *homo	China	F	(7m)	NA	+	+	36	2178	++	+	+	++	+++	+	+	-	-	-	-	-
	c.1327G>A, p.Gly433Ser; c.1516A>C, p.Thr506Pro	China	M	(16m)	+	+	+	35	2210	++	++	-	++	+++	+	-	-	-	-	-	-
	c.1327G>A, p.Gly433Ser; c.1516A>C, p.Thr506Pro	China	M	AB	+	+	NA	NA	NA	NA	NA	NA	NA	NA	NA	NA	NA	NA	NA	NA	NA
	c.1516A>C, p.Thr506Pro *homo	China	F	(49d)	NA	+	+	31	1440	++	+	+	++	+++	+	+	-	-	-	-	-
	c.1516A>C, p.Thr506Pro *homo	China	F	AB	NA	+	NA	NA	NA	NA	NA	NA	NA	NA	NA	NA	NA	NA	NA	NA	NA

	c.1516A>C, p.Thr506Pro *homo	China	F	(60d)	NA	+	+	38	2335	++	++	+	++	+++	+	+	-	-	-	-	-
Dofash et al, 2022[23]	del hg19; chr3:4272953 7-42740458; c.*152G>T	NA	M	20y	-	-	+	NA	NA	-	NA	NA	NA	+	NA	+	-	-	-	-	-
Hei Tung Lai, 2022[39]	c.1516A>C, p.Thr506Pro *homo	China	NA	(20+4w GA)	-	+	NA	NA	NA	NA	NA	showed small ears	NA	NA	NA	NA	NA	NA	NA	NA	NA
	c.1516A>C, p.Thr506Pro *homo	China	NA	(18+2w GA)	-	+	NA	NA	NA	NA	NA	NA	NA	NA	NA	NA	NA	NA	NA	NA	NA
Huang et al, 2022[40]	c.817A > T, p.Lys-273Ter; c.1516A>C, p.Thr506Pro	China	NA	(24w GA AB)	-	+	NA	NA	NA	NA	NA	NA	NA	NA	NA	NA	NA	NA	NA	NA	NA
	c.602G > A, p. Trp201Ter; c.1516A>C, p.Thr506Pro	China	NA	(24w GA AB)	-	+	NA	NA	NA	NA	NA	NA	NA	NA	NA	NA	NA	NA	NA	NA	NA
Haiming Yuan et al, 2022[41]	c.543del, p. Ser182Profs*17; c.1516A > C, p.Thr506Pro	China	F	(16d)	-	+	+	37	2250	+	+	NA	NA	+++	+	-	-	-	-	-	-
	c.602G > A, p. Trp201Ter; c.1516A > C, p.Thr506Pro	China	M	(20d)	-	+	+	35	2150	++	+	+	NA	+++	+	+	-	-	-	-	-
	c.1516A>C, p.Thr506Pro *homo	China	NA	(29w GA)	-	+	+	NA	NA	NA	NA	NA	NA	NA	NA	NA	NA	NA	NA	NA	NA
	c.1516A>C, p.Thr506Pro *homo	China	F	5y	-	+	+	31	1500	++	+	NA	++	+++	+	NA	-	-	-	-	-
	c.1516A>C, p.Thr506Pro *homo	China	NA	(28w GA)	+	+	+	NA	NA	NA	NA	NA	NA	NA	NA	NA	NA	NA	NA	NA	NA
Liu et al. 2022[42]	c.1516A>C, p.Thr506Pro *homo	China	M	(36w GA AB)	NA	+	+	NA	NA	NA	NA	NA	NA	NA	NA	NA	NA	+	-	-	-
Gurgel-Giannetti et al. 2022[43]	c.1405G>T, p.Gly469Cys; c. 1498C>T, p.Arg500Cys	Brazil	F	18m	NA	NA	NA	NA	NA	++	+	NA	++	++	+	+	-	-	+	-	-
	c.1405G>T, p.Gly469Cys; c.1498C>T, p.Arg500Cys	Brazil	F	5y	NA	NA	NA	NA	NA	++	+	NA	NA	++	+	-	-	-	+	-	-
Our case	c.1582G>A, p.Glu528Lys *homo	Italy	F	13y	-	+	+	38	2950	+	+	+	++	++	+	+	-	-	+	-	-

Abbreviations and notes: Homo, homozygous; NA, not available; F, female; M, male; d, days; w,weeks; m, months; y, years; GA, Gestational Age; AB, Abruption; abn., abnormalities; -, not present, +, present. Family History; +: positive affected sibs or spontaneous abortions, ++: positive consanguinity, +++: both positive affected sibs and consanguinity. Asphyxia/Respiratory Failure; +: present, ++: ventilator required. Facial Weakness; +: present, ++: with ophthalmoparesis. Dysphagia; +: present, ++:tube-feeding/gastrostomy required. Muscle Weakness; +: present, ++: severe, +++: no movement.
